# What Is New in Cutaneous T Cell Lymphoma?

**DOI:** 10.1007/s11912-023-01464-8

**Published:** 2023-10-24

**Authors:** Sarah Morgenroth, Andrea Roggo, Laura Pawlik, Reinhard Dummer, Egle Ramelyte

**Affiliations:** Department of Dermatology, University Hospital Zurich, University of Zurich, Zurich, Switzerland

**Keywords:** Cutaneous T cell lymphoma, Cutaneous lymphoma, Non-melanoma skin cancer, Review CTCL

## Abstract

**Purpose of Review:**

This review focuses on updates in prognosis, pathogenesis, and treatment of cutaneous T cell lymphoma (CTCL).

**Recent Findings:**

Cohort studies indicate imaging may be necessary in early-stage CTCL. Risk factors for progression of CTCL have been identified. Interactions between malignant cells and the tumor microenvironment (TME) and the skin microbiome advance the understanding of pathogenesis and tumor cell dissemination. Studies support a hypothesis of circulating malignant tumor cells. MicroRNA (miR) influence tumor progression and prognosis; the IL22-STAT3-CCL20 cascade may be a novel target. IL-4, IL-5, and IL-31 cytokines are relevant for pruritus and could be targets for therapeutic interventions. Systemic therapies, such as JAK inhibitors, targeted antibodies, and checkpoint inhibitors, show promise in advanced stages. Allogenic hematopoietic stem cell transplantation provides a potential curative option for patients.

**Summary:**

Further investigations of prognosis and translational research are necessary to improve stratification of patients for treatment.

## Introduction

**Fig. 1:**
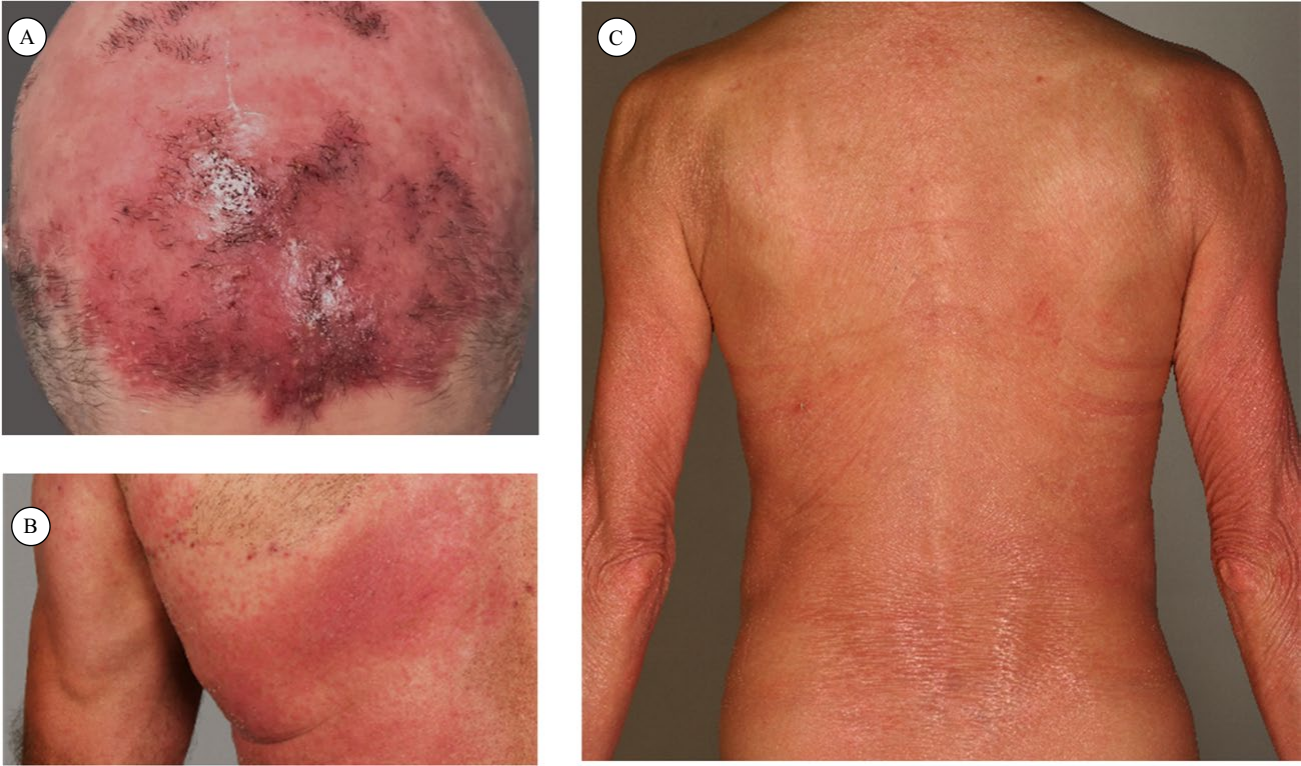
Clinical presentation in CTCL. **A** Indurated, erythematous alopecia plaques on the scalp of a patient with Sézary syndrome, **B** erythematous patches with alopecia on the scapula in a patient with mycosis fungoides, **C** erythroderma with fine scaling in a patient with Sézary syndrome

Cutaneous T cell lymphoma (CTCL) is a malignancy caused by the accumulation of neoplastic lymphocytes in the skin [1, 2]. CTCL is considered a rare disease with an incidence of 0.96 per 100,000 [3]. The European Organization for Research and Treatment of Cancer (EORTC) defines CTCL subtypes according to organ involvement, histopathological characteristics, and prognosis [1]. The main subtypes mycosis fungoides (MF), Sézary syndrome (SS), and CD30+ lymphoproliferative disorder (LPD) account for 75–80% of all cases [1]. The clinical presentation of CTCL is heterogeneous (Fig. 1). MF ranges from isolated lesions, classified as patches, plaques, or tumors, to systemic manifestation with blood involvement. The most aggressive variant SS has a leukemic presentation with Sézary cells in the blood and lymph nodes. In SS, the whole skin is usually involved, which presents clinically as a generalized erythroderma [1].

Variability of clinical presentation and diagnostic uncertainty of these rare diseases often lead to a delayed diagnosis of CTCL [4]. The diagnosis of CTCL is based on the integration of clinical presentations, histology, and molecular analysis [1]. Our understanding of the pathogenesis and prognosis of CTCL, as well as treatment options, is constantly evolving. This review will focus on recent updates in diagnosis and prognosis, pathogenesis and translational research, and established and novel treatment options.

## Diagnosis and Prognosis of CTCL

Thirty percent of patients with early-stage CTCL (IA–IIA) will progress to advanced-stage disease within 10 years of diagnosis [5]. Validated prognostic factors currently include staging of the tumor size (T), nodal (N) and systemic (M) involvements, and the extent of blood involvement (B) [6]. However, despite these prognostic factors, it is difficult to predict which 3 out of 10 patients will progress at the time of first diagnosis. A recent case series of four patients with MF and SS describes progression in an acute leukemic phase with clinical deterioration, one of which progressed from previously stable stage IA MF, highlighting this uncertainty [7].

The PROCLIPI (Prospective Cutaneous Lymphoma International Prognostic Index) study has been prospectively collecting clinical and treatment-related data since 2015 [8]. Based on longitudinal data from CTCL patients of all stages, the study aims to define a prognostic index for MF and SS [8]. A first analysis of the PROCLIPI data by Hodak et al. highlighted different strategies for initial staging of early-stage MF in the 52 centers collecting data. There is no clear consensus on imaging at first diagnosis to identify patients with clinically occult nodal metastasis. Of the 169 patients with stage IA–IB MF collected between 2015 and 2018, 45% received imaging at first diagnosis. In multivariate analysis, the clinical presence of plaques was the only relevant clinical predictor of occult lymph node involvement. Furthermore, the authors noted that clinical palpation of the lymph nodes alone appeared to be a poor predictor of lymph node involvement. In this analysis, 14% of patients imaged without clinical suspicion of lymph node involvement were subsequently upstaged from IA–IB to IIA due to occult nodal disease [9]. A further analysis aimed to identify possible risk factors to help stratify patients with a higher risk of progression when presenting with early-stage disease [10]. The risk factors identified were age > 60 years, presence of plaques, and folliculotropism [10]. Time-to-diagnosis analyses of the PROCLIPI cohort showed similar diagnostic delays in patients with early- and advanced-stage disease, indicating that first presentation with advanced disease does not correlate with longer time to diagnosis [5]. Future analyses of PROCLIPI and other registries are necessary to develop standardized prognostic factors to assist decision-making on diagnostics and therapy.

Analysis of blood involvement in MF and SS has become increasingly complex in recent years since the initial introduction of blood classification into the TNM in 2007 [11]. Initially, the blood classification B0–B2 was based on manual counts of Sézary cells [11]. In the 2018 update of the EORTC guidelines regarding blood classification and blood response in MF and SS, new recommendations for diagnosis included the detection of disease-specific changes such as increased CD4/CD8 ratio and loss of CD7 and CD26 surface markers using flow cytometry [12]. However, lack of standardization of flow cytometry between centers is an ongoing issue, and flow cytometry is still often only performed in advanced-stage lymphoma [13]. A validated standard for flow cytometry will aid clinical decision-making in evaluation for specific therapy forms, as well as response to therapy [13]. The EuroFlow consortium develops and validates standardized approaches to flow cytometry for hematologic malignancies [14]. Immunophenotypic profiling of SS patients utilizing EuroFlow multiparameter demonstrated detection of aberrant CD4+ T cell populations and identified down-regulation of novel genes THEMIS and LAIR in malignant cells compared to healthy CD4+ cells [15]. Larger analyses of this as well as other protocols are necessary to optimize standardization of flow cytometry in CTCL.

### Rare MF Subtypes

Hypopigmented MF (hMF) is a rare subtype of MF, which presents histologically with predominant infiltration of CD8+ T cells, in comparison to classic MF, which is known to be a CD4+ predominant malignancy [16, 17]. Clinically, the disease has an indolent course with a better prognosis than classic MF [18]. This behavior differs from the clinical course of other CD8+ subtypes of CTCL, such as primary cutaneous CD8+ aggressive epidermotropic T cell lymphoma with a very aggressive clinical course. The hypopigmentation has been suggested to be a surrogate marker for the antitumor immune response [19]. Cao et al. report that the dominant clonal CD8+ T cell population do not show malignant alterations in the transcriptome at the single-cell level and postulate them to be reactive. Accordingly, the authors postulate that hMF could also be a malignancy of the CD4+ T cell [20].

In folliculotropic MF (FMF), skin-resident malignant T cells accumulate in proximity of hair follicles, which clinically presents as hair loss in the affected areas [1]. The prognosis of FMF was previously considered to be similar to tumor-stage MF, with a 5-year survival rate of 66–80% [21]. Recent studies have proposed the subdivision of FMF into two prognostic subtypes [22, 23]. Early FMF presents with follicular papules and acneiform lesions; histologically, it shows subtle lymphocytic infiltrates. Advanced FMF shows thick, infiltrated alopecic plaques with deep, dense lymphocytic infiltrates [24]. Additional analyses will advance our understanding of the underlying mechanisms that lead to this difference in lymphocytic infiltration and, subsequently, prognosis.

## New Translational Findings

### Tumor Microenvironment

The tumor microenvironment (TME) includes all non-cancerous host cells; in the case of the skin, this includes keratinocytes, fibroblasts, endothelial cells, adipocytes, neurons, adipocytes, and adaptive and innate immune cells [25]. The acquisition and maintenance of the hallmarks of cancer are largely based on the interactions and communication of malignant cells with the TME [25]. Our understanding of the TME and its influence on disease progression and response to therapy in CTCL is consistently evolving. Krejsgaard et al. first proposed the term “malignant inflammation” to describe changes in the TME during the progression of CTCL [26]. A switch from a Th1-dominated reactive T cell population to a Th2-dominated inflammatory microenvironment with a reduced CD8+ infiltrate leads to suppression of cellular immunity and the antitumor response [27]. With a reduction in Th1 function, cellular immunity function decreases and T cells display markers of exhaustion and unresponsiveness [28, 29]. Recently, the interaction between malignant T cells and keratinocytes was elucidated in a study that reported decreased filaggrin and filaggrin-2 expression in lesional MF skin. Increased transepidermal water loss (TEWL) measurements mediated via STAT3 signaling suggest a skin barrier defect, which may increase the risk of bacterial infections. The authors propose the use of JAK1 inhibition in patients with advanced disease and a compromised skin barrier [30]. Further investigation of all the TME cells is needed, to identify the role and interaction of other skin resident malignant and non-malignant cells. In particular, novel techniques allow for additional spatial results; Kalliara et al. propose a workflow using single-cell spatially guided tools [31].

###  Skin Microbiome

The complexity of the TME is further compounded by the influence of exogenous antigens that interact with the local immune system of the skin on a daily basis. *Staphylococcus aureus* is a well-known cause of morbidity and mortality in CTCL patients and plays a role in the progression of the disease [32, 33]. Clinical benefit of antibiotic treatment has been shown [34]. In addition, a reduction of malignant T cell clones was noted in lesional skin before and after antibiotic treatment with decreased expression of CD25, STAT3 signaling, and cell proliferation [34]. However, an increasing prevalence (2–22%) of methicillin-resistant *Staphylococcus aureus* (MRSA) colonization is a therapeutic challenge [35]. The clinical benefit of disease control through antibiotics and the impact of antibiotic resistance needs to be further investigated in this context. Antibiotic resistance could contribute to chronic difficult-to-treat skin infections which may influence therapy response or even disease progression.

Another pathogen implicated in CTCL is *Bacillus safensis*, isolated by Dehner et al. in one recent analysis of lesional skin. In vitro co-culturing shows an induced proliferative response in cutaneous T cells. The authors hypothesize that antigenic signaling triggers the expansion of skin-homing T cells and activates regulatory bystander T cells, illustrating that the skin microbiome may play a role in the tumorigenesis of CTCL [36]. Analyses of the skin microbiome with larger sample sizes are necessary to corroborate these findings and further understand the mechanistic role of microbiomes in the development and progression of CTCL.

### CTCL Dissemination and Intra-tumor Heterogeneity

Dissemination and migration of malignant T cells in CTCL throughout the skin remain a topic of interest. Patients with primary cutaneous lymphoma typically present with multiple skin lesions. Depending on the subtype of CTCL, the percentage of patients with multiple lesions can vary. Multiple monoclonal skin lesions simultaneously indicate a constant process of recirculation of tumor cells with seeding into the skin [37].

MF displays a mature memory T cell phenotype with a monoclonal T cell receptor (TCR) in PCR analysis [16]. However, an analysis by Hamrouni et al. using next-generation sequencing of TCR in MF demonstrated TCR oligoclonality, specifically in the beta and alpha chains [38]. The authors hypothesized a primitive precursor predating the TCR gamma rearrangement, leading to the conclusion of circulating premalignant clones [38]. Iyer et al. support the hypothesis of consecutive tumor seeding through circulating neoplastic clones in an analysis using whole-exome sequencing of MF skin biopsies to detect TCR clonotypes. Furthermore, they hypothesized a recirculation of resident malignant skin resident T cells [39]. This hypothesis is supported by a finding demonstrating that resident skin resident memory T cells recirculate through down-regulation of CD69 in healthy individuals [40].

A further study by Iyer et al. using next-generation sequencing of TCR on 49 skin biopsies of MF demonstrated extensive intra-tumoral heterogeneity associated with disease stage. Reconstruction of the detected genetic aberrations using a bioinformatics approach showed a branched phylogenetic relationship pattern, supporting a model of divergent evolution of subclones over time [41]. An investigation using single-cell analysis of TCR in matched skin and blood samples of patients with leukemic MF and SS underlined the close genetic relationship within subclonal heterogeneous populations, proposing the hypothesis of continuous migration. Significantly more transcriptional activation was shown in skin-derived malignant T cells compared to matched blood–derived malignant T cells, suggesting an influence of the skin microenvironment in CTCL promoting rapid malignant expansion [42].

A recent investigation used single-cell RNA analysis and bulk whole-exome sequencing of skin samples of patients with MF and primary cutaneous anaplastic large cell lymphoma (pcALCL) to further investigate intra-tumoral heterogeneity and cancer evolution [43]. The authors propose a tumor evolution model in which a single mutated ancestor expands clonally and seeds to different anatomical sites before clinical detection of skin lesions [43]. This dissemination occurs in waves with the colonization of different areas of the skin at different points in time [43]. Another recently published report highlights the finding of two different phenotypes of the same malignant T cell clone in one patient, demonstrating phenotypic plasticity [44]. Pseudotime analysis showed transcriptomic changes during the transition between blood and skin, including adaptation to the hypoxic conditions in the skin, again highlighting the influence of the tumor microenvironment [44].

### MicroRNA (miR)

MicroRNAs (miRs) are small single-stranded RNA molecules that function as post-transcriptional regulators by inhibiting transcription or leading to degradation of messenger RNA (mRNA) [45]. miR can have an oncogenic effect or act as tumor suppressors; certain miR can act as both depending on the context and tissue [46–48]. In CTCL, miR has been reported to enhance Notch signaling, while changes in STAT signaling have been shown to influence miR expression [49, 50]. miR correlate with disease progression, prognosis, and response to treatment through regulation of many pathways previously described in CTCL [51]. Due to this interconnection of miR and signaling pathways, targeted CTCL therapies can influence miR expression. Histone deacetylase inhibitors (HDAC-I) approved for the treatment of CTCL influence the epigenetic regulation of transcription [52] which have been shown to restore the function of tumor suppressing miRs (miR-16, miR-29, miR-150, miR-26) [53]. The role of miR-26 was also investigated through the injection of miR-26a-transduced CTCL cells into immunodeficient mice [54]. This investigation demonstrated that miR-26 is a tumor suppressor associated with the inhibition of tumor invasion and metastasis via the IL22-STAT3-CCL20 cascade [54]. The authors postulate that IL-22 may be a novel target in advanced CTCL to inhibit tumor invasion and metastasis [54]. miR will continue to be the subject of many analyses, with possible implications as biomarkers, prediction of treatment outcomes, and targets of novel therapies.

### Pruritus in CTCL

Patients with advanced CTCL report pruritus with advanced-stage disease. Up to 94% of patients with the aggressive SS subtype describe severe generalized chronic itch [55, 56]. The burden of persistent pruritic feeling is often more present than the underlying disease and significantly reduces quality of life (QoL) [57, 58]. Management of severe pruritus is difficult due to poor recording in clinical trials and clinical practice [57]. In clinical practice, due to the lack of official recommendations, general antipruritic therapy such as antihistamines or anticonvulsive medication (e.g., gabapentin) is utilized [59].

An understanding of the underlying mechanism is needed to select a specific therapeutic target. In the skin, the sensation of pruritus is mediated by complex interactions of factors released and acting on the skin microenvironment that could open a therapeutic window [60]. Treatment interfering on different levels such as neuropeptides (e.g., aprepitant) or opioids (e.g., naloxone) has been reported to have a beneficial effect on itch in case reports [55]. Nevertheless, convincing results from a representative cohort are lacking.

The advanced stages of CTCL, with typical severe pruritus, shift toward a predominant Th2-cell microenvironment releasing cytokines such as IL-4, IL-5, or IL-31 that have gained attention in other itching inflammatory disorders [2, 61, 62]. First attempts to repurpose treatment with dupilumab, an antibody targeting the IL-4 receptor, led to disease progression in misdiagnosed CTCL, highlighting the challenge when interfering with the balance of immunologic defense and tolerance [63, 64].

## New Therapy Options

Treatment of CTCL is very diverse depending on the subtype and clinical presentation [6, 11, 65, 66]. The treatment of MF and SS requires a multimodal approach, including hematologists, dermatologists, and radiotherapists. The objectives of the treatment are to control symptoms, maintain quality of life (QoL), and improve survival. Treatment strategies vary from watchful waiting to skin-directed therapies (e.g., topical steroids, nitrogen mustard, phototherapy, radiotherapy), systemic therapies (e.g., immune modifiers, antimetabolites, retinoids, epigenetic regulators, monoclonal antibodies, chemotherapy), and allogeneic hematopoietic stem cell transplantation. Current treatment guidelines are available for first-line therapy according to disease stage, as well as for second-line and later-line therapy after relapse [6, 65, 67] (Tables 1 and 2).

**Table 1:** Recommended treatments according to CTCL guidelines [6, 65, 67]

Agent	Dosage	Study Type	No. of Patients (Stage)	Response rate	Disease Outcome	Drug approval
Skin directed						
Corticosteroids	Class I-III	Prospective [68]	79 (IA-IB)	88% ORR (44% CR, 44% PR)	not available	FDA* & EMA*
Bexarotene gel	0.1% / 0.5% / 1%	Phase I/II [69]	67 (IA-IIA)	63% ORR (CR 21%, 43% PR)	99 weeks (estimated median DR)	FDA
Mechlorethamine gel	0.02%	Phase II prospective [70]	130 (IA-IIA)	59% ORR (14% CR, 45% PR)	85.5% ongoing responses at 12 months	FDA & EMA
Imiquimod	5%	Compilation of case series [71]	20 (IA-IIB)	80% RR (45% CR, 35% PR)	not available	FDA* & EMA*
Local radiotherapy	-	Retrospective [72]	21 (IA)	97% CR	64% DFS at 10 years	not applicable
Psoralen ultraviolet A (PUVA)	-	Phase III [73]	45 (IB-IIA; MF)	71% ORR (22% CR, 49% PR)	9.7 months (median)	not applicable
		Review [74]	527 (all stages)	92% ORR (74% CR, 18% PR)	not available	not applicable
Narrow-band UVB	-	Review [74]	251 (all stages)	90% ORR (62% CR, 28% PR)	not available	not applicable
Low-dose Total Skin Electron Beam Therapy	-	Pooled analysis Phase II [75]	33 (IB-IIIA)	88% ORR (27% CR, 61% PR)	70.7 weeks (duration of clinical benefit)	not applicable
Systemic						
Bexarotene	6.5 mg/m2 vs. 300 mg/m2 vs. > 300 mg/m2	Phase II / III clinical studies [76]	58 (IA-IIA)	20% ORR 6.5 mg/m2, 54% ORR 300 mg/m2, 67% ORR > 300 mg/m2	73.7 weeks (median IA-IIA)	FDA & EMA
	300 mg/m2; > 300 mg/m2	Phase II / III clinical studies [77]	94 (IIB-IVB)	49% ORR (45% ORR 300 mg/m2, 55% ORR > 300 mg/m2)	42.7 weeks (median IIB-IVB)	FDA & EMA
Interferon-alpha	intramuscular/ subcutaneous / intralesional	Review [78]	304	70% ORR	not available	FDA** & EMA**
Acitretin	10-50 mg oral/day	Retrospective single center [79]	32	59% ORR (3% CR, 56% PR)	28 weeks (median)	FDA* & EMA*
Methotrexate	oral & subcutaneous	retrospective, multi-center analysis [80]	79	71% ORR	not available	FDA* & EMA*
Romidepsin	14 mg/m2 (day 1, 15, 28; 6 cycles)	pivotal, single-arm, open-label, Phase I [81]	96 (IB-IVA)	34% ORR (6% CR, 28% PR)	15 months (DOR)	FDA
Vorinostat	400 mg 1 daily	Open-label Phase IIb [82]	74(IB-IVA)	30% ORR	> 185 days (median)	FDA
		Phase III [83]	186 (IB-IVB)	5% ORR	3.1 months (PFS)	FDA
Mogamolizumab	1 mg/kg i.v.	Phase III [83]	186 (IB-IVB)	28% ORR	7.7 months (PFS)	FDA & EMA
Pegylated liposomal doxorubicin (PLD)	20mg (day 1, 15, 28)	Phase II [84]	49 (IIB, IVA, IVB)	ORR 41% (6% CR, 35% PR)	6 months (DOR)	FDA* & EMA*
	40 mg/m2 every 4 weeks	Prospective open-label trial [85]	25 (II-IV)	ORR 56% (60% SS, 50% transformed CTCL)	5 months (PFS)	FDA* & EMA*
Brentuximab vedotin	1.8 mg/kg	Phase II (open label) [86]	48 (CD30+)	73% RR (35% CR, 38% PR)	32 weeks (range: 3-93 weeks)	FDA & EMA
		Phase III [87]	66 (CD30+ MF, C-ALCL)	54.7% ORR (17.2% CR, 48.4% PR)	16.7 months (median)	FDA & EMA
Gemcitabine	1200 mg/m2 i.v.	Phase II [88]	32 (MF, peripheral TCL, PTCLU, SS)	75% ORR (22% CR, 53% PR)	10 months (DOR)	FDA* & EMA*
Pembrolizumab	2 mg/kg i.v.	Phase II [89]	24 (IB-IV)	38% ORR	65% PFS at 1 year	FDA* & EMA*
Belinostat	1000 mg/m2 i.v.	Phase II [90]	29 (IB-IVB)	14% ORR (10% CR, 4% PR)	43 days to progression (median)	FDA* & EMA*
Alemtuzumab	3/10/30 mg i.v.	Phase II [91]	22 (MF/SS)	55% ORR (32% CR, 23% PR)	12 months (median)	FDA* & EMA*
Extracorporeal photopheresis (ECP)	-	Review [92]	407 (all stages)	MRR 63% (range 31-86%); median CR 20% (0-62%)	22 months (median) (range up to 11 years)	not applicable
Allogenic HSCT	-	Prospective, matched control trial [93]	55 (IIB-IV)	not available	9 months (PFS)	not applicable
Combinations of skin-directed / intralesional / systemic therapies						
Intralesional 5-FU & Topical Imiquimod (5%)	-	Case Series [94]	9 (IA-IVA2)	100% CR		not applicable
Interferon-alpha & psoralen UVA	-	prospective randomized multicenter trial [95]	49 (IA-IIB)	80% ORR (70% CR, 10% PR)		not applicable
Interferon-alpha & Acitretin	-		49 (IA-IIB)	59% ORR (38% CR, 21% PR)		not applicable
Bexarotene & psoralen UVA	-	Phase III [73]	48 (IB-IIA; MF)	77% ORR (31% CR, 46% PR)		not applicable

*CR* complete response, *DFS* disease free survival, *DOR* duration of response, *i.v.* intravenous, *ORR* overall response rate, *PFS* progression free survival, *PR* partial response, *RR* response rate

*Approved by FDA/EMA for others indications; off-label use

**Previously approved for CTCL; agent no longer available

**Table 2:** Selected investigational agents CTCL (not yet approved)

Therapy class	Agent (dosage)	Study Type	No. of Patients (Stage)	Response Rate (ORR; CR; PR)
Skin-directed				
Resiquimod	0.03%, 0.06%	Phase I [96]	12 (IA-IB)	75% RR (33% CR, 42% PR)
PUVA with Hypericin	0.25%	Phase III [97]	169 (IA-IIA)	39% consolidated response rate
Intralesional				
TTI-621	intralesional	Phase I [98]	35 (MF, SS)	90% ORR
Systemic				
TTI-621	i.v.	Phase I [99]	29 (MF, SS)	21% ORR (3% CR, 17% PR)
Anti-KIR3DL2	0.0001 mg/kg – 10 mg/kg i.v.	Phase I [100]	44 (MF, SS)	36.4% ORR

*CR* complete response, *i.v*. intravenous, *ORR* overall response rate, *PR* partial response, *RR* response rate

In patients with early-stage disease and local tumor manifestation in the skin, the first choice is skin-directed therapy including topical corticosteroids, chlormethine gel, photodynamic therapy (PDT), and radiotherapy [101]. The main problem of skin-directed therapy is the absence of total elimination of tumor cells, with high recurrence rates after skin-directed therapy [73]. The FLASH study, a phase 3 randomized clinical trial of topical hypericin PDT, demonstrated superiority compared to placebo-controlled group in CTCL with patch and plaque lesions [97]. Radiotherapy is another option for treatment of CTCL, which can be used locally on single lesions as well as in the form of total skin electron beam radiation (TSEB) [102].

Additionally, studies have shown that skin-directed therapies do not seem to impact overall survival [103]. A recent analysis applied high-throughput T cell receptor sequencing to pre- and post-treatment biopsies of 20 lesions in patients with MF treatment with low-dose radiation therapy. Interestingly, they reported eradication of tumor clones in 5 of 16 lesions treated and reduction > 90% in the other 11 of 16 lesions. Two lesions treated with topical steroids analyzed using the same sequencing showed persistent malignant clones despite clinical improvement [103]. A current collection of clinical cases show partial to complete remission in patients with stage IIB MF after combined brentuximab vedotin and low-dose electron beam therapy, highlighting the possible advantage of combination therapies [104]. These findings highlight that further research is necessary in the combination of skin-directed and systemic therapies, which may be helpful in the simultaneous eradication of circulating blood clones and skin resident clones.

Intralesional therapy is another form of application that is often attempted. Intralesional oncolytic virotherapy using Talimogene laherparepvec (TVEC) has been shown to induce clinical response in locally advanced cutaneous lymphoma [•78]. A current phase I study investigated TTI-621, a CD17 immune checkpoint inhibitor with broad antitumor activity in relapsed and refractory hematologic malignancies, as an intralesional agent in patients with injectable CTCL [99]. TTI-621 showed activity locally as well as in adjacent and distal noninjected lesions, suggesting systemic abscopal effects [98]. Other current reports include intralesional application of immunomodulating substances, such as fluorouracil and interferon alpha [94, 105] highlighting the potential for further investigation.

In patients with systemic disease, systemic therapies are the first choice*.* Aberrant JAK-STAT signaling is common in T cell lymphoma [106]. As JAK-STAT signaling is a potential target in many T cell lymphoma, a phase 2 biomarker-driven trial investigating ruxolitinib, a JAK inhibitor, was initiated. A clinical benefit rate (complete response, partial response, or stable disease lasting at least 6 months) of 1 out of 7 patients with MF was noted [107]. Due to the small group of patients investigated, more studies are warranted.

**Fig. 2:**
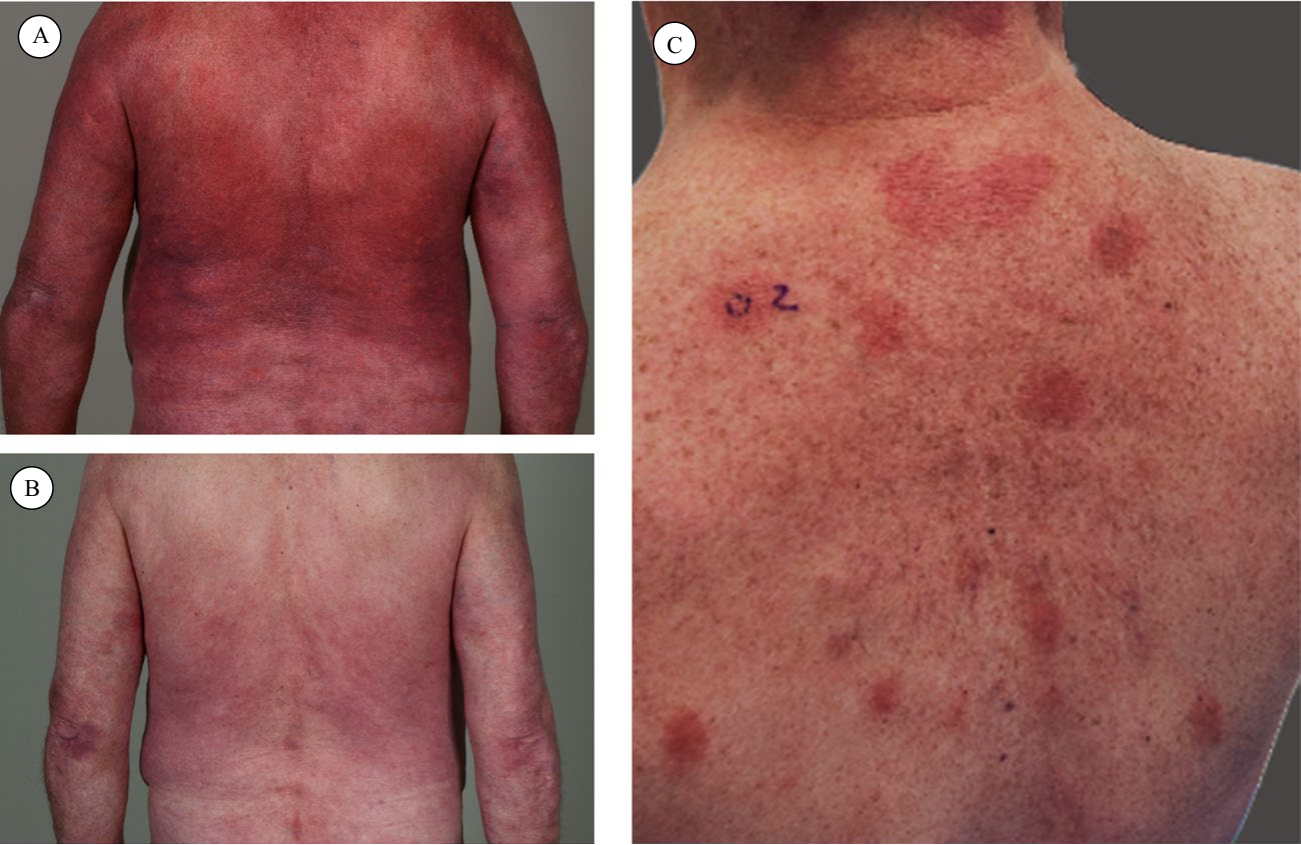
Mogamulizumab associated rash. **A** Erythroderma in a patient with Sézary syndrome before mogamulizumab, **B** clinical improvement under mogamulizumab therapy (28 infusions), **C** mogamulizumab-associated rash presenting as erythematous patches on the back

Due to the heterogeneity within CTCL with multiple relevant pathways noted previously, many different targeted therapies are in development. Mogamulizumab, a C–C chemokine receptor 4 inhibitor (CCR4), was approved after significantly prolonged progression-free survival in patients with MF and SS was shown compared to vorinostat in a phase III trial [85]. A retrospective analysis of 25 patients recently showed a significant correlation in univariate analysis of mogamulizumab-associated rash and response rate to mogamulizumab [108] (Fig. 2). Another cell surface protein targeted is KIR3DL2; results from a phase I trial show an overall response rate of 36.4% in patients with advanced MF and SS; a phase II trial (TELLOMAK) is underway [100]. Further current and novel specific targeted therapies include anti-CD30 antibody-drug conjugates, anti-CD25 antibody-drug conjugates, CD30-CD16A bispecific antibodies, and IL-2 diphtheria-toxin fusions [109].

Checkpoint inhibitors are another option for CTCL. Pembrolizumab in advanced MF and SS showed an overall response rate (ORR) of 38% in a phase II single-arm trial, with 8 of 9 patients showing ongoing responses with a median follow-up of 58 weeks [89]. However, hyperprogression has been reported in several case reports of CTCL patients treated with anti-PD1 [110]. As in other tumor types, the TME is essential to understand the tumor response to checkpoint inhibitor therapy. One recent investigation reports increased PD-1 expression and decreased PD-L2 expression on tumor cells compared to healthy CD4+ cells [91]. An analysis of patient samples before and after 3 weeks of pembrolizumab using single-cell RNA sequencing showed that response to pembrolizumab was associated with lower expression as well as expansion of CD8+ effector cells [110, 111]. Multiplexed tissue imaging and RNA sequencing were also used to investigate the response to PD-1 treatment; no differences in immune and tumor cells were found; however, topographical differences between effector PD1+, CD4+ T cell tumor cells, and immunosuppressive T cells were identified [93]. The authors developed a spatial biomarker that is strongly correlated with pembrolizumab response in CTCL [112]. Prognostic biomarkers will be necessary to correctly stratify patients for checkpoint inhibitor therapy, as ongoing responses are promising and could be relevant for the treatment of patients with advanced disease.

A further therapy option in advanced CTCL is allogeneic hematopoietic stem cell transplantation (allo-HSCT). A retrospective analysis of allo-HSCT in 113 patients with MF and SS estimated an overall survival of 38% at 5 years and progression-free survival of 26% at 5 years. Advanced phase disease and the short interval between diagnosis and transplant (< 18 months) were identified as adverse prognostic factors for progression-free survival (PFS). Advanced-stage disease and unrelated donors were identified as adverse prognostic factors for overall survival [113]. The prospective, multicenter, matched control CUTALLO trial investigated allo-HSCT in advanced CTCL. The HSCT group received reduced intensity conditioning and peripheral blood stem cells, treatment in the non-HSCT group was not standardized (investigator’s choice). The trial showed significantly longer progression-free survival in the HSCT group compared to the non-HSCT group; however, the effect on overall survival was not significant in the ITT population and in the per-protocol population. A post hoc per-protocol analysis showed a significant overall survival benefit [93]. Disease relapse after allo-HSCT can be controlled using available CTCL treatments [96].

## Conclusion

CTCL is a group of heterogeneous diseases of the skin. The pathogenesis and spread of the disease consist of numerous complex interactions between malignant and benign T cells, the innate and adaptive immune system, keratinocytes, and the skin microbiome. The accurate prediction of therapy response and survival prognosis is accordingly difficult. Further investigations regarding prognosis, as well as translational research thereof, is necessary to improve the stratification of patients for treatment.
